# Differential roles of normal and lung cancer-associated fibroblasts in microvascular network formation

**DOI:** 10.1063/5.0188238

**Published:** 2024-03-19

**Authors:** Naveen R. Natesh, Pankaj Mogha, Alan Chen, Scott J. Antonia, Shyni Varghese

**Affiliations:** 1 Department of Biomedical Engineering, Duke University, 203 Research Drive, MSRB1 Room No. 381, Durham, North Carolina 27710, USA; 2 Department of Orthopaedic Surgery, Duke University, 200 Trent Drive, Durham, North Carolina 27710, USA; 3 Department of Medical Oncology, Duke University, Durham, North Carolina 27710, USA; 4 Department of Mechanical Engineering and Materials Science, Duke University, 144 Hudson Hall, Durham, North Carolina 27710, USA

## Abstract

Perfusable microvascular networks offer promising three-dimensional *in vitro* models to study normal and compromised vascular tissues as well as phenomena such as cancer cell metastasis. Engineering of these microvascular networks generally involves the use of endothelial cells stabilized by fibroblasts to generate robust and stable vasculature. However, fibroblasts are highly heterogenous and may contribute variably to the microvascular structure. Here, we study the effect of normal and cancer-associated lung fibroblasts on the formation and function of perfusable microvascular networks. We examine the influence of cancer-associated fibroblasts on microvascular networks when cultured in direct (juxtacrine) and indirect (paracrine) contacts with endothelial cells, discovering a generative inhibition of microvasculature in juxtacrine co-cultures and a functional inhibition in paracrine co-cultures. Furthermore, we probed the secreted factors differential between cancer-associated fibroblasts and normal human lung fibroblasts, identifying several cytokines putatively influencing the resulting microvasculature morphology and functionality. These findings suggest the potential contribution of cancer-associated fibroblasts in aberrant microvasculature associated with tumors and the plausible application of such *in vitro* platforms in identifying new therapeutic targets and/or agents that can prevent formation of aberrant vascular structures.

## INTRODUCTION

Microfluidic-assisted microphysiological systems such as microvascular networks have gained widespread applications in understanding cancer biology and metastasis, drug transport, and generation of vascularized tissues *ex vivo.*^1–3^ By using microfluidic devices with unique architectures for confining three-dimensional biomaterials (e.g., hydrogels) and cell populations, it is possible to enable the generation of *in situ* capillary networks known colloquially as microvascular networks (mVNs).^4–9^ These are highly interconnected networks of endothelial cell (EC)-derived microvessels with perfusable lumens. Microvascular networks are generally formed using human umbilical-vein derived ECs (HUVECs), due to their ability to form robust networks.^4–7,10–14^ Methods to promote sprouting of HUVECs and optimize the formation of mVNs are actively being explored. First, the biomaterial used to promote vascularization is critical, with the standard models using fibrin due to its native angiogenicity and ability to degrade as the tissue forms.^15,16^ Fibrinolysis inhibitors like aprotinin are also commonly used to prevent accelerated degradation while maintaining the cellular activity and thereby enabling long-term cultures of mVNs.^17^ The presence of auxiliary cells like fibroblasts has been shown to promote robust vessel formation, and their inclusion is now routinely used in self-assembled microvascular systems.^18–20^ These cells not only promote sprouting of ECs through secretion of angiogenic factors but also structurally stabilize mVNs for long-term cultures.

Microvascular networks using the aforementioned methods have been developed to study several facets of cancer biology, such as metastasis and extravasation of immune cell types into the tumor microenvironment (TME).^12–14,21^ However, the underlying vascular networks utilized are comprised of inherently non-cancerous cells such as normal ECs and lung fibroblasts, and, thus, it is unclear the effect of cancer-associated fibroblasts (CAFs) on the formation and function of mVNs. The solid TME vasculature is characterized by high permeability, tortuosity, and blunt-endedness, leading to leaky vessels and increased interstitial fluid pressure.^22^ CAFs represent 50%–90% of the solid tumor and have been shown to influence angiogenesis in the TME through secretion of growth factors and matrix metalloproteinases.^23,24^ CAFs can also exhibit multiple nuanced functional states affected by intra-tumoral location, transcriptional profile, and interaction with other cell types of the TME including tumor epithelial cells, leading to different cell phenotypes such as inflammatory, myofibroblastic, and antigen-presenting.^25–32^

Thus far, *in vitro* systems integrating CAFs and mVNs have not been generated, with few studies measuring the effect of CAFs on angiogenesis. These studies mostly utilized tube formation or migration assays to understand basic capillary network formation.^33–38^ Moreover, these studies were static hydrogel-supported 3D cultures that were not perfusable, and, thus, only morphological characteristics of the vessel networks were assessed. Therefore, we adopt a microfluidic-assisted 3D culture to generate mVNs in an effort to understand the contribution of CAFs on vascular network formation and its functionality. Specifically, we adapted a widely used microfluidic device comprised of three hydrogel channels flanked by channels for media or other cell types.^4^ This model allowed us to probe both cell–cell communication via direct contact (i.e., juxtacrine) or indirect contact (i.e., paracrine) co-cultures of ECs with CAFs in order to study the effect of CAFs on mVNs. The findings were compared against corresponding NHLF-supported mVNs.

## RESULTS

### Co-culture of HUVECs with NHLFs in a microfluidic device

**FIG. 1.**
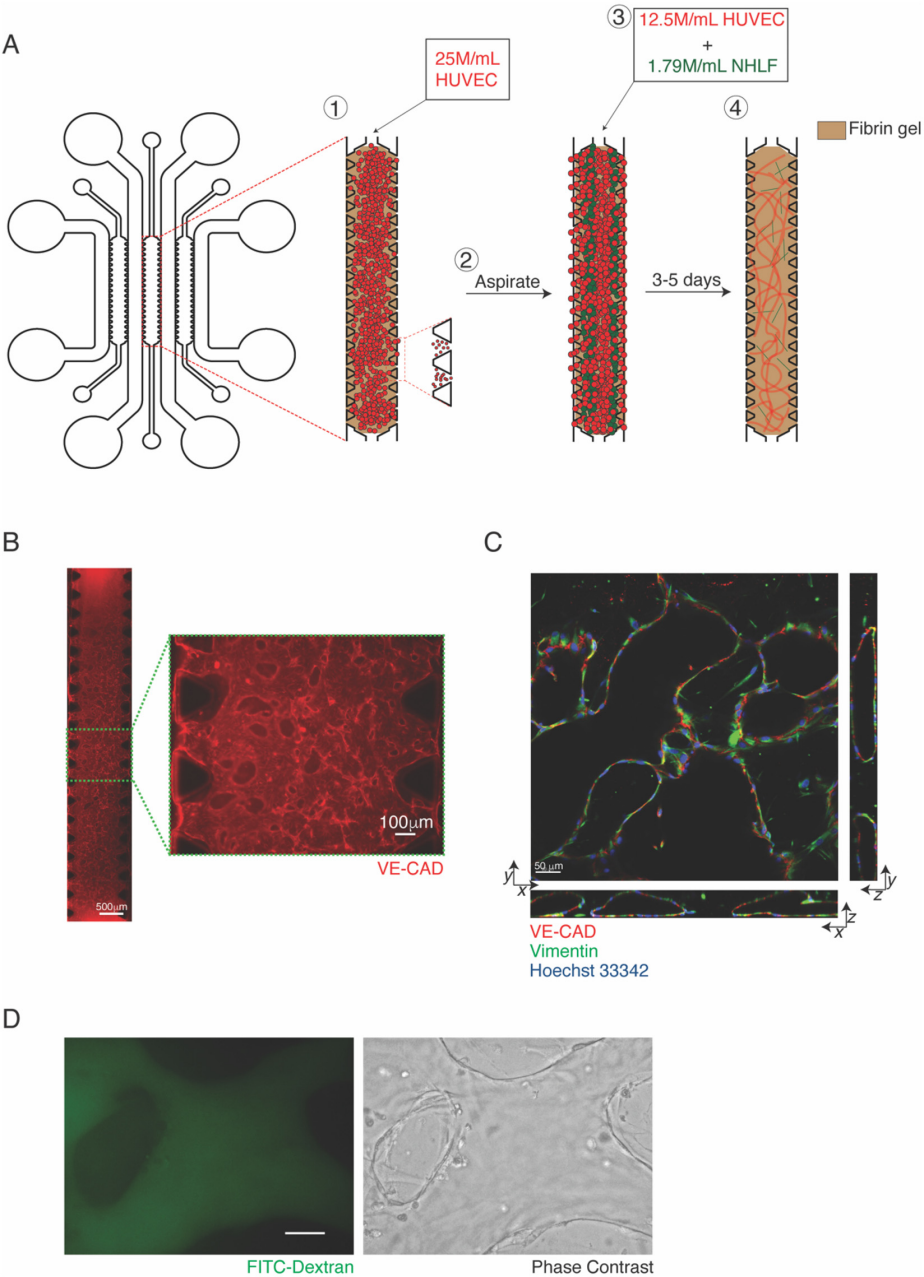
Formation of 3D microvascular network in the presence of normal human lung fibroblasts. (a) Schematic of device and incorporation of different cell populations; (1) HUVEC-laden fibrin solution is injected and aspirated to confine cells to micropost openings, (2) HUVECs and NHLFs are mixed and injected within fibrin solution and polymerized and (3) cultured for 3–5 days. (b) Representative image of full channel microvascular network with magnified image in the right. (c) 3D projection and cross sections of z-series confocal microscopy of microvascular network. (d) Representative images of FITC-Dextran perfusion through the microvessels with phase contrast image on the right; Scale bar = 50 *μ*m.

**FIG. 2.**
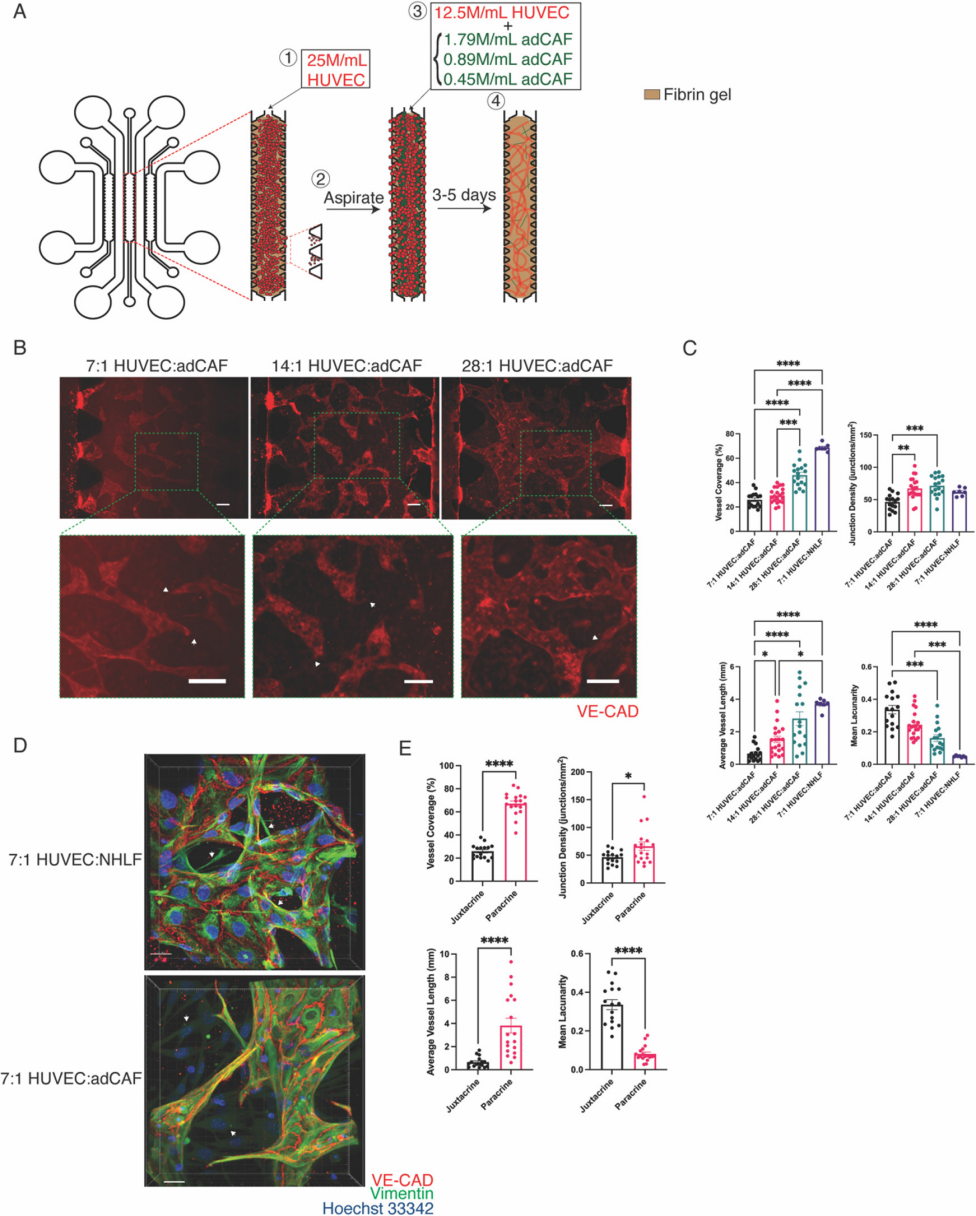
Juxtacrine co-culture of HUVECs and CAFs yield poor mVN formation. (a) Schematic of device and incorporation of cell populations with different ratios; (1) HUVEC-laden fibrin solution is injected and aspirated to confine cells to micropost openings, (2) HUVECs and adCAFs are mixed and injected within fibrin solution at different ratios, gelled and (3) cultured for 3–5 days. (b) Representative max contrast 3D projection of z-stack imaging of mVNs generated from varied density ratios of HUVEC:adCAF; green box = magnified region; Scale bar = 100 *μ*m. (c) Quantification of mVN morphology metrics between mVNs generated from varied ratios of HUVEC:adCAF and 7:1 HUVEC:NHLF; N = 6 mVNs from two devices per group, n ≥ 7 FOVs per group; Kruskal–Wallis test with Dunn's multiple comparisons. (d) Confocal microscopy z-series of mVNs; Scale bar = 20 *μ*m. (e) Quantification of mVN morphology metrics between juxtacrine and paracrine co-cultures of HUVECs and adCAFs at a 7:1 HUVEC:adCAF ratio; N = 6 mVNs from two devices (juxtacrine), N = 2 mVNs from two devices (paracrine), n ≥ 16 FOVs per group; Mann–Whitney U test. ^****^ = p < 0.0001; ^***^ = p < 0.001; ^**^ = p < 0.01; ^*^ = p < 0.05.

We employed a microfluidic device composed of three hydrogel channels interspersed with media channels. The channels are peripherally lined with evenly spaced trapezoidal microposts, which enable confinement of cell-laden hydrogels into spatially distinct regions. Using this device, we co-cultured human umbilical vein-derived endothelial cells (HUVECs) and normal human lung fibroblasts (NHLFs) at a 7:1 ratio of HUVECs to NHLFs within fibrin hydrogels [Fig. 1(a)]. Similar to prior reports, the juxtacrine co-culture of HUVECs with NHLFs resulted in robust and perfusable mVNs within 4–5 days [Fig. 1(b)]. The mVNs were characterized for various metrics that are conventionally used to describe vessel network morphology via a semi-automated software, AngioTool, which has been used extensively to characterize vascular networks both *in vitro* and *in vivo.*^5,39–42^ The mVNs displayed high vessel coverage and connectivity as measured by the frequency of vessel intersections, low lacunarity (a measure of structural non-uniformity), and consistent average vessel lengths (Table I). The presence of perfusable lumens within the engineered mVN was evident from confocal microscopy and perfusion of 2000 kDa FITC-Dextran, where the fluorescent solution was dispensed into the media inlets along the length of the mVN channel, which was confined within the microvessels [Figs. 1(c) and 1(d)]. In addition to perfusability, we also assessed vessel permeability. The mVNs exhibited relatively low permeability, with calculated coefficients similar to those reported by other studies of engineered mVNs and *in vivo* values for different mouse organ vasculatures, including breast, liver, and skin.^5,7,8,43^ The 3D confocal images suggest that NHLFs are in close proximity with the HUVECs or intersecting the vessels [white arrows in Fig. 2(d); top row].

**TABLE I.** Normal juxtacrine microvasculature metrics of functionality.

	Vessel coverage (%)	Junction density (junctions/mm^2^)	Average vessel length (mm)	Lacunarity (A.U.)	Permeability coefficient (cm/s)
Sample size	N = 6 mVNs;	N = 6 mVNs;	N = 6 mVNs;	N = 6 mVNs;	N = 6 mVNs;
	n = 7 FOVs	n = 7 FOVs	n = 7 FOVs	n = 7 FOVs	n = 15 FOVs
Mean	68.69	61.82	3.699	0.048 68	1.068 × 10^−5^
SEM	1.125	2.278	0.1258	0.001 795	2.739 × 10^−6^
Lower 95% CI of mean	65.93	56.25	3.392	0.044 29	4.809 × 10^−6^
Upper 95% CI of mean	71.44	67.40	4.007	0.053 08	1.656 × 10^−5^

### Juxtacrine co-cultures of HUVECs with adCAFs

We next examined the role of lung adenocarcinoma-derived CAFs on the formation of microvascular networks [adCAFs; Fig. 2(a)]. Given that CAFs can be heterogenous among patients as well as within the tumors, we characterized adCAFs using immunofluorescent staining and qRT-PCR (Fig. S6).^26,27^ The immunofluorescent analyses showed that the cells are vimentin-positive and αSMA-negative [Fig. S6(a)]. The minimal αSMA expression was further confirmed by qRT-PCR [Fig. S6(b)]. The gene expression analyses showed upregulation of genes implicated in the inflammatory CAF phenotype such as PDPN and CXCL12 relative to NHLF.^26^ Together, these results suggest that the adCAFs are more inflammatory-like than myofibroblastic. To examine the effect of adCAFs on mVN formation, the cells were seeded within the microfluidic device as in HUVEC-NHLF juxtacrine co-cultures but replaced NHLFs with adCAFs. Interestingly, juxtacrine co-culture of HUVECs and adCAFs at the 7:1 ratio of ECs to fibroblasts obviated microvascular network formation [Figs. 2(b) and 2(c)]. Key metrics of vascular network such as vessel coverage, junction density, average vessel length, and mean lacunarity were significantly different than those of mVNs generated using HUVECs and NHLFs [Fig. 2(c)]. The networks formed in juxtacrine co-cultures with adCAFs occupied significantly less channel area and had recurrent blunt ends, indicating a deleterious effect of adCAFs on mVN formation. We also found that the vessels of mVNs formed with adCAFs were significantly thinner than those formed with NHLFs, being limited almost to the size of single cells (∼10 *μ*m; Fig. S1). We next interrogated the spatial orientation of HUVECs and adCAFs in the mVN and compared against the corresponding mVN generated using HUVECs and NHLFs. Confocal microscopy images showed minimal proximity of adCAFs to HUVECs compared to the NHLF-supported cultures, where NHLFs were found to be in close proximity with the HUVECs and/or intersecting the vessels [white arrows in Fig. 2(d)]. The adCAFs were mostly found in the extravascular space [Fig. 2(d)]. We next decreased the number of adCAFs in the culture and examined the mVN formation in the juxtacrine co-culture [Fig. 2(a)]. The results showed that the detrimental effect of adCAFs on network formation was partially mitigated as the number of adCAFs in the culture decreased [Fig. 2(b)]. Specifically, as the HUVEC:adCAF ratio increased, a better mVN generation with higher vessel coverage and longer vessels, increased connectivity, and decreased lacunarity was observed [Fig. 2(c)]. However, even the highest ratio tested (28:1)—containing the lowest number of adCAFs—was insufficient to rescue mVN morphologies toward those generated with NHLFs. The mVNs formed in juxtacrine co-culture with adCAFs remained highly blunt-ended and were not perfusable (data not shown).

### Paracrine co-cultures of HUVECs with NHLFs and adCAFs

**FIG. 3.**
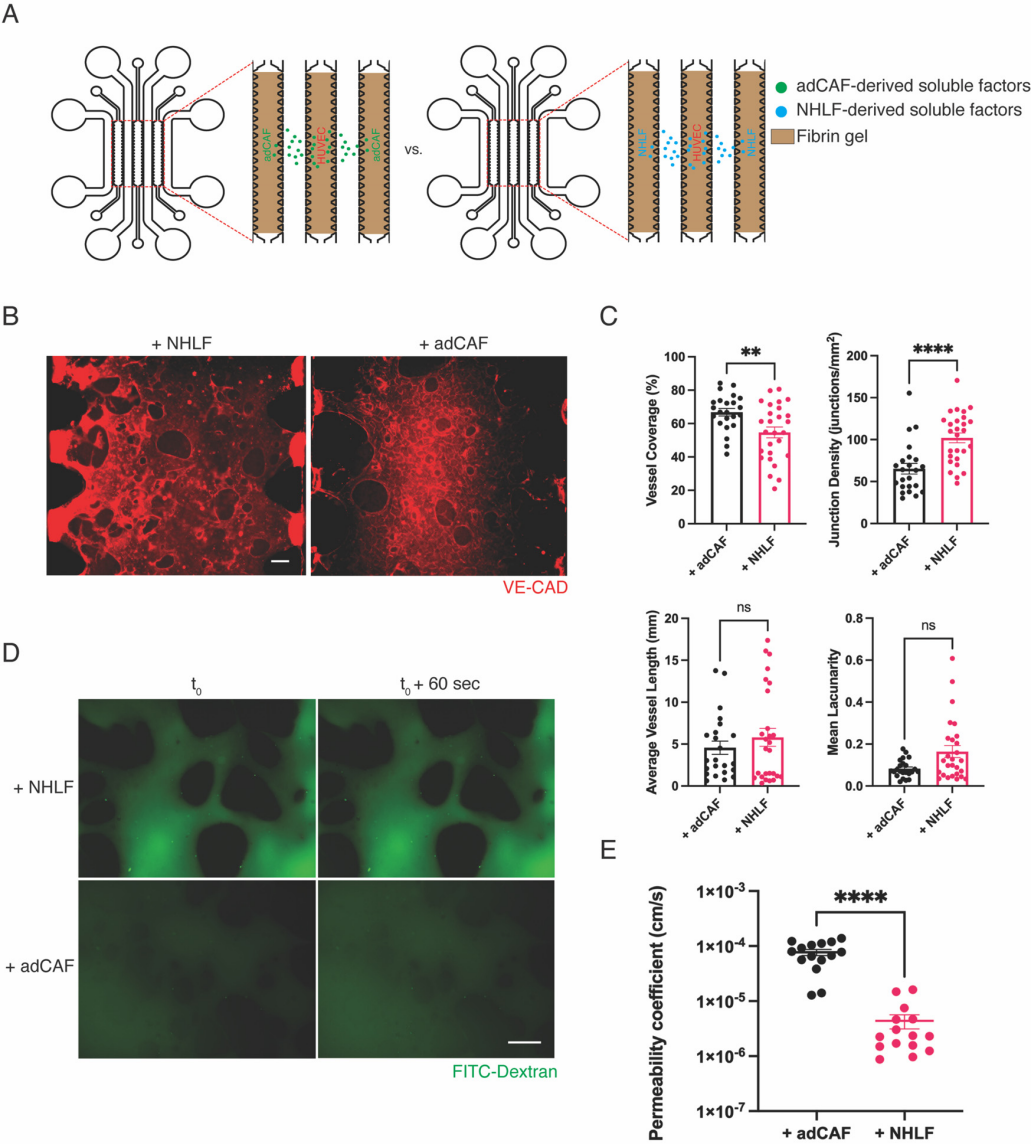
Paracrine co-culture of ECs and adCAFs yields perfusable but highly leaky mVNs. (a) Schematic of experimental design depicting shared soluble factors from paracrine co-cultures of HUVECs with NHLFs or adCAFs. (b) Representative max contrast 3D projection of z-stack imaging of mVNs; Scale bar = 100 *μ*m. (c) Quantification of mVN morphology metrics; N = 3 mVNs from three devices per group, n ≥ 23 FOVs per group; Mann–Whitney U test. (d) Representative images from 2000 kDa FITC-Dextran perfusion through mVNs over a 60 s time span; Scale bar = 100 *μ*m. (e) Permeability coefficient from mVNs formed by paracrine co-culture of HUVECs with NHLFs or adCAFs; N = 2 mVNs from two devices per group, n = 15 FOVs per group; Mann–Whitney U test. ^****^ = p < 0.0001; ^**^ = p < 0.01; Scale bar = 100 *μ*m.

Having observed that the juxtacrine co-culture had a detrimental effect on mVN formation, we carried out a paracrine co-culture of HUVECs with NHLFs or adCAFs using the initial cell ratio of 7:1 HUVEC to fibroblast. Toward this, we took advantage of the architecture of the microfluidic device by seeding HUVECs and adCAFs in spatially separated channels [Fig. 3(a)]. This configuration allowed ECs to proliferate, sprout, and anastomose within the HUVEC compartment, while adCAFs provided secreted factors from the adjacent channel. We found that paracrine co-cultures of HUVECs and adCAFs consistently formed perfusable vasculature with various morphology metrics outperforming corresponding juxtacrine co-cultures [Fig. 2(e)]. Specifically, the separation of HUVECs and adCAFs significantly increased vessel coverage, recurrence of vessel intersections, vessel length, and decreased lacunarity of the mVNs. In addition, mVNs formed from paracrine co-cultures of HUVECs and adCAFs displayed perfusable vessels, as seen through perfusion of FITC-Dextran [Fig. 3(d)].

When comparing mVNs formed from paracrine co-cultures of adCAFs against those with NHLF [HUVEC:NHLF] paracrine co-cultures, the adCAF-supported mVNs covered significantly more area, although these microvessels intersected only occasionally [Figs. 3(b) and 3(c)]. We also did not observe any significant differences in vessel length or lacunarity between the two cultures. While perfusable as compared to the juxtacrine co-cultures, the vessels of HUVEC-adCAF mVNs were highly permeable and leaky, as evident from the FITC-Dextran perfusion experiments. Permeability coefficients were found to be orders of magnitude greater in HUVEC-adCAF mVNs when compared to those from paracrine co-cultures involving HUVECs and NHLFs [Figs. 3(d) and 3(e)]. We also discovered a stark difference in paracrine vs juxtacrine co-cultures of HUVECs and NHLFs (Fig. S2). In these co-cultures, the vasculature metrics were significantly different, where paracrine mVNs covered less area, contained shorter vessels with more connections, and increased lacunarity [Fig. S2(b)]. There was also a significant decrease in permeability coefficient in the paracrine co-cultures compared to the juxtacrine co-cultures [Fig. S2(c)].

In addition to paracrine co-cultures, we also used conditioned media from NHLFs or adCAFs (NHLF-CM or adCAF-CM) and examined their effects on microvascular network formation. The mVN formed in the presence of NHLF-CM and had the largest vessels and lowest lacunarity among the different cultures [Figs. S7(a) and S7(b)]. Microvascular networks were also generated in basal EGM-2MV medium. HUVECs cultured in basal media formed microvascular networks with less vessel coverage and higher lacunarity than those formed in NHLF-CM, potentially due to the absence of NHLF-derived angiogenic factors. Similar to the co-cultures, mVNs formed in the presence of adCAF-CM showed distorted network structure. Specifically, the networks generated in adCAF-CM displayed lower vessel coverage and vessel length and increased lacunarity compared to mVNs formed in NHLF-CM. Using FITC-Dextran perfusion, we measured vessel permeability of mVNs formed by HUVECs in the various conditioned media. We found that compared to mVNs generated in NHLF-CM, vessels from adCAF-CM-cultured mVNs exhibited higher permeability [Fig. S7(c)]. The mVNs were also generally leakier in basal media than in NHLF-CM. Together, these results indicate an effect of fibroblast secreted factors on both the morphology and functionality of microvascular networks.

### Microvascular network formation from co-cultures of HUVECs and squamous cell carcinoma-derived CAFs

We next examined whether our findings using adCAFs could be extended to fibroblasts associated with other lung cancers. To this end, we used lung CAFs derived from squamous cell carcinoma (sqCAFs)—a rarer but more malignant non-small cell lung cancer. Akin to adCAFs, juxtacrine co-cultures of HUVECs and sqCAFs showed a trend of insufficient mVN formation (Fig. S3). The morphology of mVNs was increasingly normalized upon decreasing the amount of sqCAFs relative to HUVECs in the culture [Fig. S3(a)]. Similar to juxtacrine co-cultures involving adCAFs, the mVNs containing sqCAFs were highly blunt-ended, and decreasing the amount of sqCAFs in the juxtacrine co-cultures did not completely rescue the mVN formation [Figs. S3(a) and S3(b)].

However, paracrine co-cultures of sqCAFs and HUVECs rendered perfusable mVNs, though morphologies of the networks were vastly different than those formed with adCAFs [Figs. S4(a), S4(b), and S5]. Vessels from cultures containing sqCAFs were more tortuous, lower in lacunarity, and highly intersecting, as opposed to those generated from cultures with adCAFs, which displayed much larger and less frequently intersecting vessels, with larger extravascular gaps [Figs. S4(a) and S4(b)]. However, perfusion of the mVNs with FITC-Dextran suggested no significant difference in permeability between the two CAF-supported mVNs [Figs. S4(d) and S4(d)].

### Identification of secreted factors of HUVEC-NHLF vs HUVEC-adCAF paracrine co-culture

**FIG. 4.**
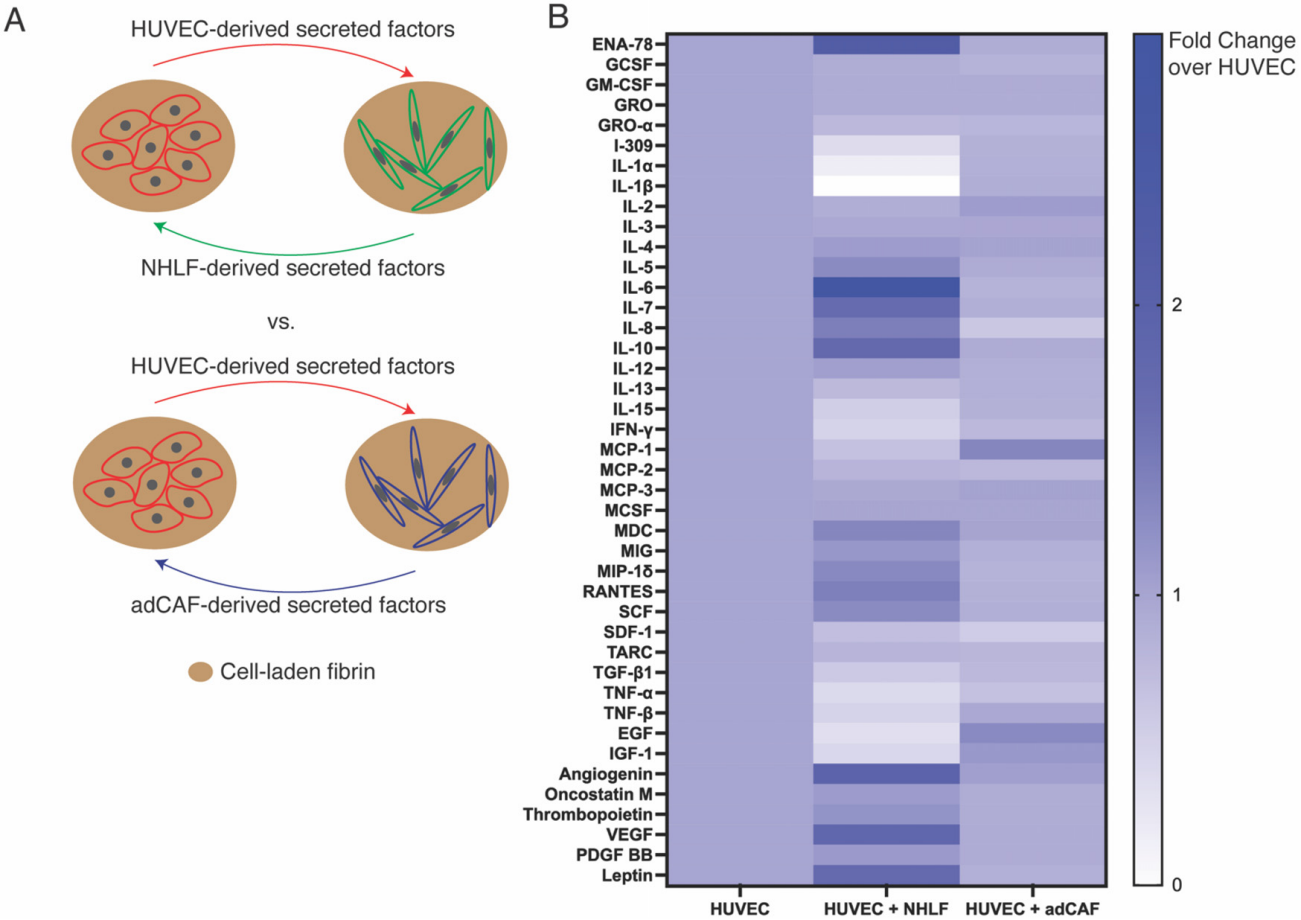
Secretome analysis of paracrine co-cultures of ECs and CAFs identify several soluble factors differentially secreted between co-cultures with CAFs vs NHLFs. (a) Schematic depicting paracrine co-culture of cell-laden fibrin releasing secreted factors in shared media. (b) Heatmap illustrating fold change of secreted factors released from indicated paracrine co-cultures, relative to monocultured HUVECs.

In order to identify the secreted factors from adCAFs and NHLFs, we used a targeted cytokine antibody array to evaluate their differential levels in each culture condition [Fig. 4(a)]. Toward this, HUVEC-laden fibrin gels were cultured in isolation or co-cultured with either adCAF- or NHLF-laden fibrin gels. We found a number of differentially secreted factors between the HUVECs and adCAFs and HUVECs and NHLFs co-cultures [Fig. 4(b)]. Notably, co-cultures with adCAFs yielded more pro-inflammatory molecules such as IL-1β, IFN-γ, MCP-1, TNF-α/β, and TGF-β1. In contrast, co-cultures involving NHLFs contained markedly higher amounts of angiogenic factors such as VEGF, Leptin, and Angiogenin.

## DISCUSSION

Normal human lung fibroblasts are widely used in engineered mVN models, where they serve to structurally stabilize network vessels for longer term cultures as well as secrete angiogenic growth factors that encourage network formation and promote functionality.^4–6,13,18–20^ In the present study, we examined how cancer-associated fibroblasts (CAFs) would affect the formation and functionality of microvascular networks *in vitro*. We found that CAFs in juxtacrine co-culture with HUVECs attenuated vascular network formation and did not result in perfusable vessels. Lowering the number of CAFs resulted in better mVNs morphologically resembling closely to those generated using HUVECs and NHLFs, albeit non-perfusable. In contrast, paracrine co-cultures of HUVECs with CAFs not only yielded perfusable mVNs higher in vessel coverage and connectivity but also displayed higher permeability than those involving NHLFs, recapitulating the formation of characteristic leaky vascular networks similar to those seen in the TME.^22^

Regarding the differences in paracrine co-cultures, the cytokine array analysis involving either NHLF or adCAFs showed distinct changes in secreted factors between the two conditions. Notably, we observed an increase in inflammatory cytokines secreted in HUVEC:adCAF co-cultures such as IL-1β, IFN-γ, and TNF-α/β. These pro-inflammatory cytokines could contribute to the changes in the mVN formation and function. Previous studies have shown that these inflammatory cytokines can increase the amount of reactive oxygen species and lead to leaky vessel formation in animal models.^44–48^ MCP-1, or CCL2, also appeared higher in the HUVEC:adCAF paracrine co-cultures, which, in addition to inflammation, is also implicated in promoting angiogenesis and might explain the larger vessel coverage observed in HUVEC:adCAF paracrine co-cultures.^49,50^

Impaired microvascular networks associated with CAFs could be due to a multitude of factors. CAFs are known to remodel the extracellular matrix (ECM), which could hinder HUVEC motility, rendering merging and anastomosis to create perfusable vascular networks challenging.^51–55^ Indeed, we found that in juxtacrine co-cultures, CAFs tended to situate away from microvessel HUVECs and instead mostly occupy the extravascular space. These, in conjunction with CAF secreted factors, could explain the attenuated mVN formation in juxtacrine co-cultures, since titrating down the number of CAFs resulted in mVNs morphologically resembling mVNs generated using HUVECs and NHLFs. The inflammatory phenotype is also suggested by the secretome in the co-culture involving adCAFs compared to that involving NHLFs. The inflammatory phenotype of the auxiliary cells could have a significant effect on the formation and function of the mVN.

Although direct comparison of the secretomes of NHLFs and lung CAFs is challenging and has not been reported, there has been some investigation into the secretome of NHLFs cultured in fibrin gels and lung CAFs from a number of patient tumors.^26,56^ The NHLFs used in this study largely match with the secretome of NHLFs cultured in fibrin gels by Akinbote *et al.*, except for VEGF and Angiogenin, and this difference could be due to donor-to-donor differences, or due to the presence of HUVECs in the co-culture.^56^ In contrast, the secretome of HUVEC:adCAF co-cultures displayed higher levels of pro-inflammatory molecules as described earlier. Consistent with the secretome, the characterization of the adCAFs implicates it to be more inflammatory-like, resembling the recently discovered ADH1B+ CAFs from Grout *et al.*^26^ It is well-known that the secretome of CAFs can be highly influenced by the tumor microenvironment, such as hypoxia or different tumor cell interactions influencing the secretome and even inducing transitioning of CAFs into different phenotypes.^30,57^ Furthermore, CAFs are highly heterogeneous based not only on the original organ but also on the donor, the stage/grade of the tumor, and the spatial localization within the tumor.^23,26,27,57–59^

Between the two CAF populations, we observed a similar deleterious effect on the formation of mVNs in cultures with HUVECs. However, while the two CAF-supported mVNs formed in paracrine co-cultures yielded similar trends in vasculature metrics such as vessel coverage, sqCAF-supported mVNs were observably more tortuous and intersecting and lesser in lacunarity than those from cultures with adCAFs. Although we did not probe the differential secretome between the two subtypes, we expect there to be differences given the phenotypic difference between mVNs formed. There is a growing appreciation into the differential prognosis of lung adenocarcinoma and lung squamous cell carcinoma (LUSC) as it has been demonstrated that LUSC is more malignant and usually caught later in the transformation process.^60^ The contribution of CAF to the TME between the cancer subtypes is also of interest, and differential biomarkers have been identified between the two, potentially motivating better stratification and therapy options for patients.^61–63^ Therefore, our findings underscore the differences in emergence and functionality of mVNs in the presence of adCAFs and sqCAFs, highlighting the need to continually consider cancer subtype and biological context when designing or testing oncology therapeutics.^64^ A prior study by Sewell-Loftin *et al.* showed improved vascular network formation when breast CAFs were cultured with HUVECs in fibrin gels.^33^ The authors attributed the findings to the greater contractility of the CAFs. While breast CAFs enhanced vascular network formation compared to normal breast fibroblasts, NHLF-supported mVNs were evidently more robust. This indicates that between cancers, the fibroblasts will have a differential impact on mVN formation, and in our studied case of the lung, there was a deleterious effect in juxtacrine co-cultures when comparing NHLF to lung CAF. In fact, we found that VEGF secretion was markedly lower from lung CAFs, as opposed to higher secretion from breast CAFs in the aforementioned study. In addition, prior studies have shown the differential ability of NHLFs to promote perfusable mVN formation based on the donor.^65^ While our study examined the effect of different fibroblasts (e.g., normal vs cancer-associated fibroblasts), it does not capture donor-to-donor variability in either NHLFs or CAFs as we used the respective fibroblasts from individual donors. Hence, it is difficult to generalize the findings, as there are transcriptional and phenotypical differences of fibroblasts between individuals. Future studies which investigate the effects of CAFs and NHLFs from multiple donors will, therefore, be necessary to extend our findings. Moreover, the differences seen between CAFs and NHLFs could also be attributed to the subtypes of CAFs, as the CAFs used in the current study are more iCAF-like than myCAF-like; the latter subtype could have a different effect on microvascular networks. However, regardless of the origin of the CAFs studied (adCAF vs sqCAF), our results showed a significant role of CAFs in influencing mVNs through comparing both NHLF-supported cultures and those involving either CAF subtype.

## CONCLUSION

In the present study, we investigated the influence of CAFs on the formation and functionality of microvascular networks. We discovered a generative inhibition of mVNs in the presence of CAFs in juxtacrine co-cultures and an emergent leaky vasculature in paracrine co-cultures. Delving into the cancer subtypes, we found correspondingly higher permeability in the adCAF- and sqCAF-supported mVNs when compared to those involving NHLFs, yet stark phenotypic differences in all engineered mVNs. Finally, we identified cytokines differentially secreted in adCAF- and NHLF-supported cultures, putatively explaining the morphological differences observed in the microfluidic device. This work suggests a potential role for CAFs in affecting underlying morphology and functionality of leaky vascular networks such as those observed within the tumor.

## METHODS

### Cell culture

HUVECs were purchased from Lonza (cat. no. CC-2519) and maintained in EGM-2MV media (cat. no. CC-3202). NHLFs were purchased from Lonza (cat. no. CC-2512) and maintained in FGM-2 fibroblast growth media (cat. no. CC3132). Human tumor-associated fibroblasts derived from lung adenocarcinoma (cat. no. HC-6013A) or lung squamous cell carcinoma (cat. no. HC-6013S) were purchased from Cell Biologics and maintained in Fibroblast Medium (cat. no. M2267) supplemented with 5% v/v FBS on Collagen Type 1-coated (Corning; cat. no. 354236) 100 mm tissue culture plates.

### Device design and Fabrication

The microfluidic devices were designed as previously reported.^4^ The device consists of three 8.4 × 1.3 mm^2^ channels for the cell culture that can be confined by trapezoidal microposts and each apposed to media channels of 1 mm width and 100 *μ*m height. As in the original publication, the microposts are 0.037 mm^2^ isosceles trapezoids composed of a 300 *μ*m large base and 70 *μ*m small base connected by lines forming a 60° angle with the large base. Microposts are separated by 100 *μ*m. Inlet and outlet ports of media and cells have diameters of 4 and 1 mm, respectively.

Devices were fabricated as described earlier.^4^ In brief, SU-8 100 photoresist (Kayaku Advanced Materials) was spin-coated onto 100 mm silicon wafers. Next, a 20k dpi film photomask with the device design (designed on AutoCAD) was exposed with 10 mW/cm^2^ intensity UV light onto the wafer for 45 s. The exposed wafer was then developed with SU-8 developer. The patterned wafer was treated in a vacuum chamber with trichloro(1*H*,1*H*,2*H*,2*H*-perfluorooctyl)silane (Millipore cat. no. 448931) for 15 min at 25 °C. PDMS and curing agent (Sylgard 184, Dow Corning, Michigan, USA) were mixed at 10:1 weight ratio and cast onto the SU-8 master wafer and baked at 60 °C for 3 h. Patterned PDMS was separated from the wafer, and inlets and outlets were punched out with 1 and 4 mm biopsy punches. PDMS was bonded to 70% ethanol-cleaned glass coverslips after 1 min of oxygen plasma treatment. Channels were then coated with 1 mg/ml poly-(D)-lysine (Sigma-Aldrich cat. no. P7886), incubated for 4 h at 37 °C, subsequently washed with DI water, and dried at 60 °C to recover hydrophobicity. Devices were used within 72 h of drying.

### Cell seeding within the device

HUVECs were trypsinized and resuspended in either 1 U/ml Thrombin:EGM-2MV (Thrombin from bovine plasma, Sigma cat. no. T6634-500UN) or 3.96 U/ml Thrombin:EGM-2MV at concentrations of 50 or 25M/ml, respectively. NHLFs or CAFs were added to 25M/ml suspensions of HUVECs at different cell density ratios. 50M/ml suspensions of HUVECs were mixed with equal volumes of 5 mg/ml fibrinogen (bovine, Thermofisher cat. no. J63267-03), injected, and quickly aspirated from the device to confine HUVEC cells to the micropost openings. Next, equal volumes of 25M/ml HUVEC:fibroblasts and 5 mg/ml fibrinogen were mixed and injected into the device. Fibrin gels were formed at 25 °C in a humidified chamber for 15 min. EGM-2MV media was flushed through the channels and added to each media inlet. Media was replaced daily. All cells used were between passages 3 and 6, and the passage number was concordant between the different cells in each experiment. Microvascular networks were assessed after 96 h of culture.

### Fibroblast conditioned media experiment

NHLFs or adCAFs were cultured in their respective growth media until 70% confluence. Media was then changed to EGM-2MV, and cells were cultured for 24 h before collecting the medium, which was centrifuged at 15 000 RPM for 15 min and stored at −20 °C until use. HUVECs were seeded into every hydrogel channel within the microfluidic device, as described earlier. Devices were maintained in 1:1 Conditioned Media:EGM-2MV for four days before analysis.

### Immunofluorescence

Devices were fixed in 4% paraformaldehyde for 30 min at 25 °C and washed in 1X PBS. Devices were then permeabilized for 20 min and blocked for 2 h at 25 °C. Primary antibody (VE-CAD; R&D cat. no. AF938, Vimentin; Sigma-Aldrich cat. no. V6630) was added to devices and incubated overnight at 4 °C. Devices were then washed in 1X PBS + 0.1% Tween-20 (Wash Buffer). Secondary antibody was added to devices and incubated overnight at 4 °C. Devices were then washed using Wash Buffer and subsequently imaged on a Keyence BZ-X710 Fluorescence Microscope or Andor Dragonfly Spinning Disk Confocal Plus microscope. For immunofluorescence of adCAFs, cells were grown on collagen type 1-coated 15 mm diameter glass coverslips until 70% confluent and then fixed in 4% PFA for 15 min at 25 °C. Cells were then permeabilized for 10 min and blocked for 1 h at 25 °C. Cells were stained with primary antibody at 4 °C overnight and stained with secondary antibody for 1 h at 25 °C. Coverslips were mounted onto glass slides using ProLong™ Diamond Antifade Mountant with DAPI (Invitrogen cat. no. P36971). Antibodies used are described in the supplementary material, File 1.

### Microvasculature morphology metrics

Max-contrast z-stack projections were created from z-stacks of vasculature and loaded into AngioTool v0.6a.^66^ AngioTool allows semi-automated quantification of vessel parameters such as average vessel length, mean lacunarity, and junction density, and we processed each image as described in the NCI user manual (see https://ccrod.cancer.gov/confluence/display/ROB2/Quick±Guide). All other analyses were performed using ImageJ (v2.14.0).

### Microvasculature permeability assay and permeability coefficient quantification

To measure permeability, 10 *μ*l 2000 kDa FITC-Dextran (Sigma cat. no. FD2000S) was added to appropriate media inlets in order to create a weak pressure drop across the microvasculature networks. Fluorescent images were captured every 30 s for 5 min. In ImageJ, analysis windows including part of extravascular space and across vessels of diameter less than 50 *μ*m (to ensure circularity) were created, and fluorescent intensity was measured. Permeability coefficient was calculated based on the following equation:^67,68^

$$P\left(\frac{cm}{s}\right)=\frac{1}{\left(I_{i}-I_{b}\right)}\left(\frac{I_{f}-I_{i}}{\Delta t}\right)\times \frac{d}{4},$$

where $I_{b},\;I_{i}$, and $I_{f}$ represent average intensity of the measuring window in the background, initial time point, and final time point, respectively. Δt is the duration between initial and final time points in seconds, and d is the average vessel diameter in the measuring window. N = 15 were obtained per group.

### Secretome analysis

NHLF or adCAFs were dissociated and resuspended in 3.96 U/ml Thrombin:EGM-2MV at a concentration of 3.32M/ml. Equal volumes of NHLF/adCAF suspension and 5 mg/ml Fibrinogen were mixed, and 100 *μ*l was dispensed into the 24-well plate well surface. Fibrin was gelled at 37 °C for 15 min. Next, HUVECs were dissociated and resuspended in 3.96 U/ml Thrombin:EGM-2MV at a concentration of 50M/ml. Equal volumes of HUVEC suspension and 5 mg/ml Fibrinogen were mixed, and 20 *μ*l droplets were dispensed onto 0.4 *μ*m pore size polycarbonate membrane Transwell inserts (Corning cat. no. CLS3413) in a 24-well plate. Fibrin was polymerized at 37 °C for 15 min. Transwell inserts containing HUVECs were carefully placed into wells with NHLF/adCAF, and 600 *μ*l EGM-2MV media was added to each well. Cultures were maintained for five days with serum-free media replacement on day 3. Supernatant was collected, spun at 15 000 RPM for 5 min at 4 °C, and stored at −20 °C until use. Supernatants were subjected to the Human Cytokine Antibody Array (Abcam cat. no. ab133997), according to the manufacturer's instructions.

### RNA isolation and RT-PCR

Total RNA was isolated from adCAFs or NHLFs grown in EGM-2MV media for 24 h using Trizol reagent (Invitrogen cat. no. 15596026). Reverse transcription was performed using the iScript cDNA Synthesis Kit (Bio-Rad cat. no. 1708890). Quantitative Real-time RT-PCR using iTaq Universal SyBR Green Super-Mix (Bio-Rad cat. no. 1725124) was performed with a CFX96 Touch PCR instrument (Bio-Rad cat. no. 1855196). Glyceraldehyde 3-phosphate dehydrogenase (GAPDH) was used as a control housekeeping gene. Primers used are described in the supplementary material, File 1.

### Statistics

For all comparisons in which the data were normally distributed, t-tests or one-way ANOVAs were used to test differences in groups. Kruskal-Wallis tests were performed for comparisons of more than two groups, with Dunn's multiple comparison testing. Welch corrections were performed not assuming equal variance between groups. Mann–Whitney U-tests were performed for datasets in which normal distribution was not observed. A p-value less than 0.05 was considered statistically significant. Statistics and plotting were performed in GraphPad Prism 9.

## SUPPLEMENTARY MATERIAL

See the supplementary material for several data, including adCAF characterization, and morphology and functionality of microvascular networks in the presence of sqCAFs. Also included is the list of primers used for qRT-PCR analysis and all antibodies used in the study.

## Data Availability

The data that support the findings of this study are available from the corresponding author upon reasonable request.
